# Oxybutynin-Induced Hyperthermia in a Patient With Parkinson’s Disease

**DOI:** 10.7759/cureus.14701

**Published:** 2021-04-26

**Authors:** Saad Ahmad, Jonathan Vincent M Reyes, Joseph Lieber

**Affiliations:** 1 Internal Medicine, Icahn School of Medicine at Mount Sinai, Elmhurst Hospital Center, Elmhurst, USA; 2 Internal Medicine/Nephrology, Mount Sinai Elmhurst Hospital, New York City, USA

**Keywords:** parkinson’s disease, heat stroke, anticholinergic, hyperthermia, oxybutynin, thermoregulation, perspiration

## Abstract

Impaired thermoregulation and heat intolerance may be intrinsic to autonomic dysfunction in Parkinson’s disease due to disturbances in perspiration regulation. Thermoregulatory impairment leading to hyperthermia/heatstroke can be accentuated with the usage of anticholinergics, which block the ability to sweat. Oxybutynin chloride is one of the most used anticholinergic agents in clinical practice for the management of detrusor hyperreflexia secondary to neurogenic bladder dysfunction and is often used in the setting of Parkinson’s disease. We present a rare instance of oxybutynin-induced heatstroke in an elderly patient with Parkinson’s disease.

## Introduction

The presence of a wide array of non-motor symptoms indicates that Parkinson’s disease is not restricted to the substantia nigra or central dopaminergic system [1]. There are still debates on whether the disease originates peripherally or simply works in parallel peripherally. Morphological studies have demonstrated Lewy bodies in sympathetic trunk ganglia, cardiac nerve cells, intestinal plexus, and other areas of the peripheral nervous system [2]. Regardless of the origin of the pathology, there is no denying that the presence of autonomic failure causes a great impact on the patients’ daily function and quality of life [1]. Autonomic regulatory disorders were long considered to be a late manifestation of the disease process, however, this is no longer the case given significant evidence of autonomic dysfunction occurring prior to diagnosis [2]. Involvement of the autonomic nervous system contributes to dysfunctions in cardiovascular, urinary, sexual, gastric, and thermoregulatory systems.

Over one-third of Parkinson’s disease patients can be assumed to have a thermoregulatory dysfunction with perspiration-related symptoms. Hyperhidrosis manifested as excessive sweating primarily affecting the axillae, face, palms, and soles of the feet, and hypohidrosis, a lack of sweating, have been described in current literature in patients with Parkinson's disease [3]. However, between the two perspiration-related symptoms, hyperhidrosis has been reported as a more common non-motor manifestation. Topical agents used to treat focal hyperhidrosis include aluminum chloride hexahydrate and anticholinergics [4]; oxybutynin has been proven effective in some trials, although not primarily used for this purpose [5]. The etiology behind hyperhidrosis in Parkinson’s disease is still in question as some studies suggest hyperhidrosis in axial body parts could be the expression of compensatory mechanisms for a defective submotor outflow, likely due to poor cholinergic activation in sweat glands [6].

The most used definition of heat stroke is Bouchama’s definition which defines heatstroke as a core body temperature that rises above 40 °C, often accompanied by dry skin and central nervous system abnormalities [7]. Bouchama has also stated that heatstroke is a form of hyperthermia associated with a systemic inflammatory response leading to multiorgan dysfunction. Individuals with impaired thermoregulatory sweating due to neurological disease states may be at greater risk of symptomatic anhidrosis from taking drugs with anticholinergic activity; this includes medications such as oxybutynin [8]. Peripheral blockade of muscarinic receptors in exocrine sweat glands would further contribute to impaired heat dissipation, potentially leading to the development of hyperthermia or heatstroke [9].

## Case presentation

A 71-year-old female with a past medical history significant for Parkinson's disease presented to the emergency room with a fever and altered mental status. History of the patient was obtained from the daughter due to the patient's impairment on presentation. According to the daughter, the onset of the symptoms was first noted in the morning when the patient’s husband went to wake her up. The patient was warm and non-responsive to her family; the patient’s daughter took her oral temperature which read 107 °F, at which point the family contacted emergency medical services (EMS). As per the daughter, the patient started taking oxybutynin extended-release 5 mg oral, once daily, roughly five days prior to presentation to help with her overactive bladder. The daughter had reported that the mother had “doubled up” on some doses to alleviate her urinary symptoms. There were no reported alleviating or exacerbating factors to the patient’s condition. These symptoms never occurred prior. The patient did not complain of acute pain nor did the daughter state any knowledge of perceived pain. There was no reported history of recent travel for anyone in the family or a history of active tuberculosis. There was no prior history of hospitalizations or surgeries, drug allergies, or usage of tobacco, alcohol, or illicit substances. According to the daughter, at baseline, the patient requires assistance with basic activities due to tremors; however, the patient is generally alert and oriented with independent ambulation. The family had stated that the patient was not eating or drinking much lately. Aside from the oxybutynin, home medications included carbidopa/levodopa 192 mg, two capsules every four hours.

**Figure 1:**
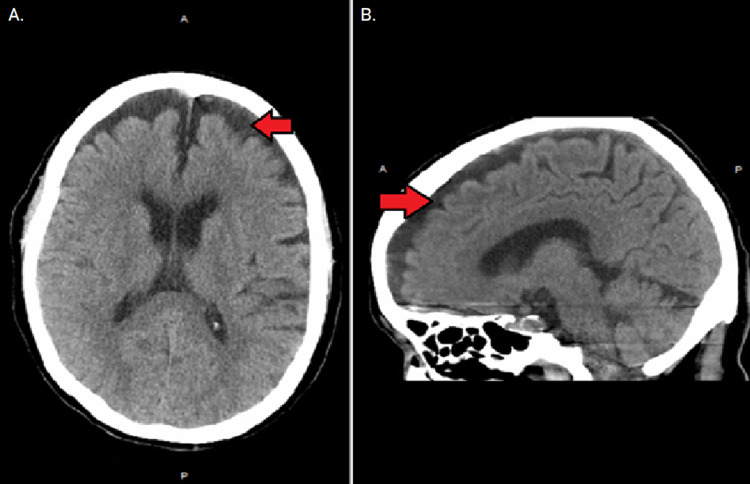
Bilateral frontal subdural hygromas (A) Axial view and (B) sagittal view.

Upon arrival to the emergency department, it was noted that the patient had an oral temperature of 106.5 °F (41.4 °C), heart rate of 145 beats/min, respiratory rate of 27 breaths/min, blood pressure of 133/87 mmHg, and oxygen saturation of 100%. The initial examination revealed that the patient was stuporous, and her skin was dry and warm to the touch. There were no signs of rigidity and her pupils were equal, round, and reactive to light bilaterally. Cardiac, respiratory, and abdominal examinations were unremarkable. Laboratory results demonstrated hyponatremia (128 mEq/L), hypokalemia (3.3 mEq/L), elevated creatine (1.32 mg/dL) with an unknown baseline, and slightly elevated lactate (2.4 mmol/L). Hematology, thyroid function, and liver function tests were normal. The blood gas demonstrated a pH of 7.482, carbon dioxide (CO_2_) of 17.6 mmol/L, and bicarbonate (HCO_3_) of 18 mmol/L. Serum creatine phosphokinase was elevated at 316 U/L, while initial magnesium and calcium concentrations were normal. Cerebrospinal fluid analysis and urinalysis were unremarkable. A portable chest X-ray and a CT head scan without contrast demonstrated no acute findings; however, imaging did demonstrate small, approximately 1 cm, bilateral frontal subdural hygromas (Figure 1). The patient’s bioreference test was negative, indicating no presence of COVID-19.

The patient was upgraded to the medical intensive care unit (MICU) where her temperature was decreased to normal levels using external whole-body cooling with a cooling blanket, room temperature intravenous NaCl 0.9% infusions, and acetaminophen. The patient remained in the MICU without demonstrating leukocytosis or growth in blood, urine, sputum, and cerebrospinal fluid cultures. The patient received three days of vancomycin and cefepime which were later discontinued due to no known source of infection. Throughout the course of the MICU, the patient’s electrolytes were replenished, her oxybutynin was held and she was placed on deep vein thrombosis (DVT) prophylaxis with heparin.

On the medical floors, the patient required multiple straight catheterizations due to urinary retention, as bladder scans showed 600 to 1200 mL of urine. A foley catheter was placed after urology consultation recommendation.

Upon discharge, the patient was instructed to refrain from taking oxybutynin and to follow up with urology for possible alternative management.

## Discussion

Heatstroke is a common cause of hyperthermia and approximately 80% of deaths associated with heatstroke occur in individuals older than 50-years-old [10]. A normal body temperature is maintained at approximately 98.6 °F (37 °C) by the medial preoptic area/anterior hypothalamus through mechanisms of thermoregulation. The hypothalamic temperature-regulating center senses the temperature of the blood perfusing the brain and activates peripheral processes, predominately perspiration-related mechanisms [11]. These perspiration-related mechanisms, although effective at first, eventually cause further salt and water loss, impairing thermoregulation. Paired with shunting of blood from the central circulation to the skin and muscles, the reduced central volume leads to impaired visceral perfusion and subsequent organ failure [11]. It is important to note that most patients recover fully after a short period of hyperthermia, but patients exposed to higher temperatures for extended periods of time are more susceptible to progression to multi-organ failure and possible death. Hyperthermia, even if mild and occurring for a short period of time, may cause cognitive impairment and affect attention, memory, and information processing. Elevated temperatures have been linked to increased brain blood barrier permeability contributing to cerebral edema. Additionally, increased temperatures have been linked to decreased mitochondrial metabolism, and in the absence of elevated lactate levels, this may indicate a reduction in cerebral metabolic activity; this could manifest as cognitive and neurological symptoms [12].

As mentioned before, blood flow to central organs such as the gastrointestinal (GI) tract, is reduced in the setting of hyperthermia causing damage to cell membranes, protein denaturation, and increased production of free radicals. Overall, this loss of GI barrier integrity allows for the translocation of gut bacteria and/or endotoxins. This can lead to a systemic inflammatory cascade and multi-organ damage. Animal models have demonstrated improved survival with the initiation of antibiotics in the setting of heatstroke [12]. Although our patient did not demonstrate such, it is an important consideration for the treatment of other patients with a more septic-like picture.

Dysfunctions in the perspiration-related mechanisms can also predispose individuals to hypercoagulability as there is significant evidence demonstrating a link between hyperthermia and changes in coagulation. Coagulation activation was seen in human subjects with an exercise-induced elevation of the core body temperature and therapeutic hyperthermia; an increase in body temperature from 37.6 °C to 39.4 °C was associated with increased thrombin generation and impaired endogenous fibrinolysis [13]. There is accumulating evidence that extracellular DNA and DNA binding proteins, such as histones, released from nucleosomes of degraded cells may form a surface on which activated coagulation factor complexes can be assembled. Histones have been shown to activate platelets and stimulate thrombin generation. Activation and binding of neutrophils by DNA components have been shown to result in the formation of neutrophil extracellular traps which have been shown to further activate coagulation by proteolytic cleavage of physiological anticoagulants by abundant neutrophilic elastases [13]. The susceptibility to a hypercoagulable state further emphasizes the need for DVT prophylaxis in these patients.

The risk of heatstroke is increased when homeostatic mechanisms are compromised by drugs that impair perspiration or promote dehydration. These drugs include diuretics, neuroleptics, or medications with anticholinergic properties. Oxybutynin chloride is a tertiary amine compound with anticholinergic properties and is a commonly used drug known for its musculotropic relaxant activity [14]. Relative selectivity of the M3 and M1 parasympathetic muscarinic receptors allows for antispasmodic activity responsible for its relaxant effects on the detrusor muscle in many conditions such as Parkinson’s disease, allowing for increased bladder capacity and decreased frequency of uninhibited bladder contractions. Of the five types of muscarinic acetylcholine receptors, the M3 receptor is the predominant receptor subtype found in eccrine sweat glands [15]. This makes oxybutynin’s selectivity of the M3 receptor of particular importance when it comes to thermoregulation as the agent has demonstrated to be an effective alternative treatment for hyperhidrosis, which can be primary or secondary in origin [5]. Acetylcholine binds to G protein-coupled cholinergic muscarinic M3 receptors in the basolateral membrane of clear cells, activating a cascade of intracellular pathways that causes an influx of extracellular Ca+2 [8] which then leads to myoepithelial cell contraction and sweat production through subsequent efflux of K+ and Cl- ions and isotonic egress from the luminal side of the clear cell; isotonic fluid becomes hypotonic following active NaCl reabsorption in the sweat duct. A blockade of M3 receptors with the usage of oxybutynin would cause anti-perspiration effects leading to a predisposition for hyperthermia/heatstroke. This is of particular importance as some studies have demonstrated that hyperhidrosis secondary to peripheral or central nervous system abnormalities in axial body parts could lead to the expression of a compensatory mechanism for a defective submotor outflow in hands and feet. Noninvasive recordings of the galvanic potentials of the skin via the sympathetic sudomotor skin reflex response (SSR) demonstrated a lower prevalence of an N-type response waveform in Parkinson’s disease, pointing to a diminished cholinergic innervation of sweat glands [6]. This raises the question of safety when using anticholinergics in patients with Parkinson’s disease, especially individuals over the age of 50 years. The lack of acetylcholine release peripherally via an M3 blockade and poor submotor outflow puts patients at greater risk for hyperthermia and heatstroke. This was likely the mechanism that caused our patient to exhibit heatstroke.

As effective as oxybutynin, countless studies have demonstrated a significant increase in maximum plasma concentration in elderly patients with significant comorbidities, indicating a greater susceptibility for adverse events. Epidemiological studies have reported rates of 44-60% anticholinergic drug use in the elderly in an inpatient setting and 9-27% in an outpatient setting [8]. The elderly population is especially vulnerable to potential drug side effects as the elimination time of oxybutynin could be increased due to poly-pharmacy, resulting in drug interactions and/or competitions for the cytochrome P-450 CYP3A4 enzyme [8]. However, this was not the situation with our patient. It is important to know that the extended-release form allows for steady plasma concentrations by minimizing the peak and trough concentrations seen with twice-daily formulations. The difference between the two formulations is likely due to the lack of N-desethyl-oxybutynin metabolite formation in the extended-release form [14]. Most adverse events related to the administration of conventional oxybutynin have been attributed to this active metabolite which has demonstrated a high affinity for muscarinic receptors in animal models [14].

Despite the anticholinergic adverse events, these “peripheral” anticholinergics are marketed as first-line treatment for overactive bladder; oxybutynin, administered orally or via a transdermal patch, is the most studied out of all these agents. Other effective agents include tolterodine, solifenacin, and darifenacin [1]. The efficacy of other agents, such as alpha-adrenoreceptor antagonists, is not supported by a high level of evidence [16]. Although oxybutynin is linked to adverse reactions (such as constipation, headaches, dry mouth, thermoregulatory dysfunction), the timing, dosage, and route of administration are key to the effective and optimal management of patients with avoidance of adverse effects.

As mentioned before, the medial preoptic area/anterior hypothalamus is responsible for controlling the core temperature at which sweating commences. This thermoregulatory set point is finely regulated and subject to the influence of numerous medications. Given the juxtaposition of the medial preoptic area to the suprachiasmatic nucleus of the hypothalamus, there is a circadian rhythm of body temperature in which the threshold for the onset of sweating is lowest around 2 am [17]. The timing of agents such as oxybutynin in relation to this circadian rhythm, as well as differences in bioavailability and pharmacodynamic half-life dependent on formulations, may result in variable alteration in an individual sweating response. In this case, the timing in which the patient had taken her medication may have predisposed her to heatstroke. As mentioned before, the extended-release allows for steady plasma concentrations by minimizing the peak and trough concentrations seen with twice-daily formulations. Usage of the extended-release formulation would be the safer option in elderly individuals and would less likely interfere with the circadian rhythm of temperature regulation if given at an appropriate time. However, it is important to note that the patient did double her medication dosage without proper medical advice. Timing along with increased dosage, even if it was the extended-release, could have been a contributing factor to the patient’s overall presentation.

Regarding routes of administration, a large, randomized double-blinded study compared transdermal oxybutynin to a placebo. The transdermal route of oxybutynin was associated with reduced micturition frequency and increased voided volumes. Side effects of the transdermal form were primarily related to the site of administration. Dry mouth, and other common side effects associated with the oral formulation, were less common in the application of the transdermal formulation [18]. Although the oral route is the most thoroughly evaluated and commonly used form, this trial and others shed light on the possibility of using the transdermal route to reduce or avoid major adverse reactions.

The patient’s electrolyte abnormalities could have been contributed by thermal dysregulation, oxybutynin usage, and poor diet. The patient’s blood gas demonstrated the presence of a likely acute respiratory alkalosis in the setting of hyperventilation with metabolic compensation. A possible explanation for the abnormal blood gas could be hyperthermia-induced hyperventilation to help cool the elevated temperatures detected by the brain [19]. There was also a slight elevation of creatine phosphokinase noted upon initial lab draw, likely due to the elevated temperatures.

It is important to note that this case had occurred during the month of July on a particular day that was hot and humid. These weather conditions could have been a contributing factor to the patient’s overall presentation. In addition, patients older than 65 years are at an increased risk for a heat-related illness especially with insufficient fluids and anticholinergic usage.

## Conclusions

Conditions that affect thermoregulation, such as Parkinson’s disease, are known to lead to a predisposition for hyperthermia and heatstroke. Heatstroke accounts for significant morbidity and mortality in those who have not been treated promptly. It is critical to initiate cooling and observe patients for end-organ damage. The risk of heatstroke is increased when homeostatic mechanisms are compromised by drugs that impair perspiration. Drugs with anticholinergic properties, such as oxybutynin, are among these medications. Clinicians should use caution and warn patients about the dangers of using drugs that could impair thermoregulation. Effective teaching can lead to proper usage of the medication and help prevent the adverse effects of hyperthermia and heatstroke. Different routes of administration, dosages, and timings of administration for the medication should be explored and adjusted to fit the needs of the patient.
